# Suicidal ideation among Chinese cancer inpatients of general hospitals: prevalence and correlates

**DOI:** 10.18632/oncotarget.15350

**Published:** 2017-02-15

**Authors:** Bao-Liang Zhong, Si-Heng Li, Shu-Yan Lv, Shun-Li Tian, Zhi-Dong Liu, Xu-Bin Li, Hong-Qing Zhuang, Ran Tao, Wei Zhang, Chuan-Jun Zhuo

**Affiliations:** ^1^ Affiliated Wuhan Mental Health Center, The Ninth Clinical School, Tongji Medical College of Huazhong University of Science & Technology, Wuhan, Hubei Province, People's Republic of China; ^2^ First Clinical Medical College & School of Basic Medical Science, Peking University, Beijing, People's Republic of China; ^3^ Department of Psychiatry, Tianjin Mental Health Center, Tianjin Anding Hospital, Tianjin, People's Republic of China; ^4^ Department of Healthcare, Tianjing Medical University General Hospital, Tianjin, People's Republic of China; ^5^ Department of Psychiatry, Tianjin Fourth Central Hospital, Tianjin, People's Republic of China; ^6^ Department of Radiology, Tianjin Medical University Cancer Institute & Hospital, Tianjin, People's Republic of China; ^7^ Department of Psychology, General Hospital of Beijing Military Region, Beijing, People's Republic of China; ^8^ Department of Psychiatry, Wenzhou Seventh People's Hospital, Wenzhou, Zhejiang Province, People's Republic of China

**Keywords:** cancer, inpatient, suicidal ideation, prevalence, correlate

## Abstract

Cancer patients are at high risk for suicide, particularly when they are informed about the cancer diagnosis or hospitalized for cancer treatment. Therefore, oncology healthcare settings such as large general hospitals in China, may represent an ideal setting to identify and treat suicidality in cancer patients. However, the clinical epidemiology of suicidality of Chinese cancer patients remains largely unknown. This study examined the prevalence and correlates of suicidal ideation among Chinese cancer inpatients of large general hospitals. A total of 517 cancer inpatients were consecutively recruited from two tertiary general hospitals of a metropolitan city in northern China, and administered with standardized questionnaires to collect data on sociodemographics, mental health, and cancer-related clinical characteristics. Suicidal ideation and mental health were measured with a single self-report question “In the past month, did you think about ending your life?” and Hospital Anxiety and Depression Scale, respectively. The one-month prevalence of suicidal ideation was 15.3% in Chinese cancer inpatients. In multivariable Logistic regression, depression, anxiety, moderate-to-severe pain, metastatic cancer, poor performance status, surgery, and palliative care were significantly associated with suicidal ideation. Cancer inpatients of large Chinese general hospitals have high prevalence of suicidal ideation and therefore potentially at high risk for suicide. Suicide prevention efforts for cancer inpatients should include periodic evaluation of suicidality, effective pain management, psychooncological supports, and, when necessary, psychiatric treatment and crisis intervention.

## INTRODUCTION

In China, the incidence of cancer is increasing rapidly in recent years and cancer has become the leading cause of mortality for the Chinese population, with 4.3 million newly diagnosed cancer patients and 2.8 million deaths due to cancer in 2015 [1, 2]. Because of the advances in early detection and treatment of cancer and the very large number of Chinese population, quite a great number of people are now living with cancer in China and the number of cancer survivors is expected to increase in upcoming years [1].

Living with cancer is challenging, because patients often have to experience an array of physical and psychological problems, including loss of appetite, pain, fatigue, depression, despair, fear, and even suicidal feelings. Accumulating evidence suggests that the risk of attempted and completed suicide among cancer patients is much higher than among the general population, particularly among those who had recently received a cancer diagnosis or whose cancer recurs [3–5]. Further, although cancer patients’ risk of suicide may decrease over time, the elevated suicide risk in patients can still persist for many years after the diagnosis and treatment of cancer [6]. In addition, studies also indicate that other types of non-fatal suicidal behaviors, i.e., desire for hastened death and suicidal thoughts and plans, are fairly common in cancer patients [7, 8].

Suicide is not only a tragedy for individuals who lose the battle with cancer and their entire families, friends, and significant others, but also a major source of stress for their healthcare providers who have made great efforts to treat their illness [9]. Findings from a Chinese study that investigated the effects of patient suicide on nursing staff also revealed a decline in quality of nursing care among those who recently experienced patient suicide [10]. Therefore, how to effectively recognize and manage cancer patients who are suicidal or at risk for suicide is a critical issue in clinical oncology practice.

Sociocultural factors play an important role in the occurrence of suicidal behaviors [11]. Unlike people of Western countries, Chinese people often keep cancer a secret and those with cancer are reluctant to report their cancer-related emotional distress and suicidal feelings to medical professionals, due to stigma associated with cancer in the Chinese society; for example, some patients believe that cancer is a punishment from God for their past mistakes [12, 13]. Importantly, the subspecialty healthcare for addressing psychosocial problems of cancer patients, psychooncology services, have not been provided in most of the Chinese general hospitals and cancer specialty hospitals [13, 14]. Due to the lack of training in clinical psychiatry, Chinese physicians do not routinely screen their patients for mental health problems and suicidality [15, 16]. Furthermore, even if some physicians of Chinese large hospitals are capable of recognizing depressed/suicidal individuals, most of them are usually too busy to pay adequate attention to patients’ emotional and suicidal issues. Results of a survey conducted among cancer inpatients of a university-affiliated hospital in Beijing, China, demonstrated a very high prevalence of major depression in Chinese cancer patients, but only 6.9% of the patients with major depression were detected or referred for psychiatric consultation [13]. Considering the strong association between untreated major depression and suicidality [17], it is very likely that suicidal problems in cancer patients are prevalent but seriously neglected in China's hospitals.

Health services utilization studies have shown that over two-thirds Chinese cancer patients prefer to seek treatment from oncology departments of large general hospitals (i.e., secondary and tertiary hospitals) [18, 19]. Given the high risk of suicidality in cancer patients, large Chinese general hospitals may represent an ideal setting to identify and treat suicidality in cancer patients. Understanding the prevalence and clinical characteristics of suicidality in cancer patients may facilitate cancer-specific suicide prevention efforts. However, to the best of our knowledge, only one published study has examined the epidemiology of suicidal ideation (SI) in Chinese cancer patients [20]. Because the sample of this study was restricted to gynecological cancer patients of one tumor hospital, its findings may not be generalized to a wider population of cancer patients as well as cancer patients of general hospitals. Therefore, the epidemiology of suicidality of Chinese cancer patients in large healthcare settings remains largely unknown.

SI is a broad term that refers to thoughts of engaging in any suicide-related behavior, ranging from transient and intermittent thoughts about death and more severe rumination and creation of a plan to kill oneself [5, 21]. Although only a minority of SI individuals attempt suicide and only a minority of attempters die, many studies suggest that SI predicts later attempted and completed suicide [22–25]. As a result, any expression of SI such as talking about wanting to die or to kill oneself is one of the most common warning signs of suicide [26]. Thus, determining the epidemiology of SI in clinical oncology settings would lend important insights into the early identification of suicidal patients. This study was set out to investigate the prevalence and correlates of SI in Chinese cancer inpatients treated in large general hospitals. The hypothesis of this study was that SI would be prevalent in Chinese cancer inpatients, and it would be associated with a number of socio-demographic, psychological, and cancer-related characteristics.

## RESULTS

The mean age of the 517 subjects who completed the survey was 59.7 years (standard deviation=11.7, range=21-97), and 50.7% were females. The 517 completers and 218 non-completers were comparable in proportion of females (50.7% vs. 46.3%, χ2=1.159, P=0.282) and age (59.7±11.7 vs. 61.8±20.0, t=1.774, P=0.076). The most common types of cancer were lung (28.6%), digestive tract (23.2%), and breast cancer (15.1%). No subjects endorsed a personal history of mental illness. Table 1 shows the socio-demographic and clinical characteristics of the participants.

**Table 1:** Characteristics of subjects and prevalence rates of suicidal ideation by variables

Variables		No. of inpatients	No. of suicidal ideators	Rate of suicidal ideation, %	χ2	P
Gender	Male	255	33	12.9	2.127	0.145
	Female	262	46	17.6		
Age (years)	20-49	111	19	17.1		
	50-64	240	31	12.9	1.94	0.379
	65+	166	29	17.5		
Education (years)	≤6	126	16	12.7		
	7-9	206	28	13.6	3.833	0.28
	10-12	147	26	17.7		
	≥13	38	9	23.7		
Marital status	Married or remarried	489	72	14.7	2.16	0.142
	Others*	28	7	25.0		
Place of abode	Urban	305	39	12.8	3.573	0.059
	Rural	212	40	18.9		
Living arrangement	Not alone	464	68	14.7	1.367	0.242
	Alone	53	11	20.8		
Religious belief	No	492	70	14.2	8.712	0.003
	Yes	25	9	36.0		
Self-rated economic status	Moderate or good	312	39	12.5	4.699	0.030
	Poor	205	40	19.5		
A family history of psychiatric illness	No	503	72	14.3	13.399	<0.001
	Yes	14	7	50.0		
A relative or friend who completed/attempted suicide	No	505	74	14.7	6.607	0.010
	Yes	12	5	41.7		
Depressive symptoms	No	321	14	4.4	77.982	<0.001
	Yes	196	65	33.2		
Anxiety symptoms	No	132	2	1.5	25.945	<0.001
	Yes	387	77	20.0		
Intensity of pain	None and mild	363	35	9.6	29.929	<0.001
	Moderate and severe	154	44	28.6		
Cancer staging	Local	145	9	6.2		
	Regional	144	22	15.3	15.089	0.001
	Metastatic	228	48	21.1		
Time since cancer diagnosis (months)	≤18	275	51	18.5	4.838	0.028
	>18	242	28	11.6		
No. of hospital admissions	≤2	117	21	17.9	0.832	0.362
	>2	400	58	14.5		
ECOG Scale score of performance status#	1-2	395	41	10.4	31.055	<0.001
	3-4	122	38	31.1		
Current treatment regimen	Chemotherapy or radiotherapy	338	40	11.8		
	Palliative care	143	29	20.3	10.205	0.006
	Surgery	36	10	27.8		

* “Others” included never married, separated, cohabitating, divorced, and widowed.

#1=Restricted in physically strenuous activity but ambulatory and able to carry out work of a light or sedentary nature, e.g., light house work, office work; 2=Ambulatory and capable of all self-care but unable to carry out any work activities; up and about more than 50% of waking hours; 3= Capable of only limited self-care; confined to bed or chair more than 50% of waking hours; 4= Completely disabled; cannot carry on any self-care; totally confined to bed or chair.

The one-month prevalence of SI was 15.3% in the whole sample, with 12.9% in males and 17.6% in females. SI prevalence rates by other socio-demographic and clinical characteristics are shown in Table 1. The results of comparisons between subgroups according respondent characteristics showed that SI patients were more likely to have religious belief, poor financial status, a score of 3-4 on the Eastern Cooperative Oncology Group (ECOG) Performance Status Scale, a family history of psychiatric illness, a relative or friend who completed/attempted suicide, depressive symptoms, anxiety symptoms, moderate-to-severe pain, advanced cancer, a short duration after cancer diagnosis, poor performance status, and a current treatment regimen of palliative care or surgery.

The prevalence of SI in inpatients according to the site of cancer are listed in Table 2. SI rates differ significantly by cancer site (χ2=13.075, P=0.023), with highest rate in breast (21.8%), followed by gynecological (20.9%) and liver cancer (20.8%).

**Table 2:** Prevalence of suicidal ideation by types of cancer

Cancer site	No. of inpatients	No. of suicidal ideators	Rate of suicidal ideation (%)
Lung cancer	148	22	14.9
Digestive tract cancer	120	12	10.0
Breast cancer	78	17	21.8
Gynecological cancer	67	14	20.9
Liver cancer	53	11	20.8
Others*	60	3	5.0

* “Others” included 15 thyroid, 10 urinary system, 5 head and neck, 5 prostatic, 5 brain and 10 other cancer.

Multivariable logistic regression analyses (Table 3) revealed that depression, anxiety, moderate-to-severe pain, metastatic cancer, poor performance status, surgery and palliative care were independently and positively associated with SI among cancer inpatients.

**Table 3:** Multivariable logistic regression of correlates for suicidal ideation of cancer patients

Factor	Risk level	Reference level	Coefficient	Standard error	Wald χ2	P	OR(95%CI)
Depressive symptoms	Yes	No	1.857	0.338	30.226	<0.001	6.41(3.30,12.42)
Anxiety symptoms	Yes	No	1.936	0.759	6.512	0.011	6.93(1.57,30.66)
Pain intensity	Moderate and severe	None and mild	0.853	0.294	8.434	0.004	2.35(1.32,4.17)
Cancer stage	Metastatic	Local	1.077	0.442	5.938	0.015	2.94(1.26,6.98)
ECOG Scale score of performance status	3-4	1-2	0.699	0.303	5.322	0.021	2.01(1.11,3.64)
Current treatment regimen	Surgery	Chemotherapy or radiotherapy	1.890	0.540	12.245	<0.001	6.62(2.30,19.07)
	Palliative care	Chemotherapy or radiotherapy	0.643	0.327	3.867	0.049	1.90(1.01,3.61)

## DISCUSSION

In the context of the poor physician-patient relationship and incomplete medical liability insurance system in today's China [27, 28], a patient's suicide death in healthcare settings may lead to a serious conflict between the healthcare providers and relatives of the patient, and the hospital often has to lose a large sum of money to smooth the anger and sadness of the patient's relatives. Therefore, suicide prevention in inpatients with cancer (and other illnesses) would benefit both patients and administrators of Chinese general hospitals. Investigating the clinical epidemiology of suicidal behaviors is the first step towards suicide prevention.

To the best of our knowledge, this is the first study in China to examine the prevalence and correlates of SI in cancer patients of large general hospitals. The main finding of this study is that 15.3% cancer inpatients of Chinese large general hospitals in the Tianjin area reported having SI in the previous month. This prevalence is higher than that reported among cancer outpatients of a regional cancer center in United Kingdom (7.8%) [7], among breast cancer patients undergoing surgery in Korea (10.9%) [29], among prostate cancer survivors in the United States (12.4%) [30], and among terminal cancer outpatients in Japan (8.6%) [31]. The prevalence we found in Chinese cancer inpatients is similar to that reported in other cohorts, including the 17.7% prevalence in a population-based sample of American cancer patients [32], the 15% prevalence in Japanese patients with unresectable lung cancer in Japan [33], and the 18.1% prevalence in Chinese gynecological cancer patients [20], but lower than that reported in Portuguese cancer patients referred for psychiatric consultation (34.6%) [34] and Korean stomach cancer survivors (34.7%) [35]. The prevalence variations across these studies may be partly due to the disparities in sampling methods, heterogeneity in the samples (i.e., patients with one type of cancer only vs. various types of cancer), definitions of SI (i.e., desire for hastened death vs. thoughts of ending one's life), assessment instruments (i.e., suicidal item of a depression scale vs. Beck Scale for Suicide Ideation), and the study settings (i.e., outpatient vs. inpatient). However, the SI prevalence in our study is still higher than the pooled prevalence found in three meta-analyses of Chinese population-based surveys (general population: 3.9%, older adults: 11.5%, and college students: 10.7%) [36–38].

On the whole, our results on correlates of SI in cancer inpatients are substantially different from those of general population, because, in the final multivariable logistic regression model, no sociodemographic factors known to be risk factors for SI in the general population were kept, and we failed to replicate findings on most general factors of SI in the general population (i.e., a family history of psychiatric illness and exposure to suicide of a relative or friend). Only psychological and cancer-related clinical factors remained in the final model, which is consistent with some [30, 33, 35], but not all previous studies [7, 20, 29, 32]. This may be related to the particular clinical setting of this study–inpatient departments of large general hospitals, where patients were recently diagnosed with cancer or admitted for cancer treatment, because studies have found that patients are at particularly high risk for severe emotional and physical distress at the time of disclosure of the cancer diagnosis and treatment [3, 6]. Therefore, psychological and cancer-related clinical factors may prevail over other factors in their relationships with SI at this study setting.

A systematic review and meta-analysis reported that approximately 50% of the Chinese cancer patients have clinically significant depressive or anxiety symptoms [39]. In the present study, we further found that depression and anxiety were two most significant contributing factors to SI, with ORs of 6.41 and 6.93, respectively. This finding is a little different from what is generally acknowledged, that is, depression is the most significant contributing factor for suicidality in the general population [40, 41]. This difference could be partly attributed to the high level of psychological stress due to cancer diagnosis or treatment in these cancer patients.

Similar to earlier findings [5, 7, 33], SI in cancer patients was associated with moderate-to-severe pain. In the literature, the association between pain and elevated risk of SI is very complex. A survey reported that pain, especially prolonged or uncontrolled pain, was the most common reason for SI of cancer patients who considered suicide a reasonable/justifiable future option [42]. Pain also may influence the risk of suicidality through some mediating mechanisms, for example, pain may cause depression and sleep disturbance, which in turn increase the risk of SI [43].

Cancer sites are also reported to be associated with increased risk of suicidality in some previous studies [6, 20]. Similarly, we found statistically significant differences in SI prevalence rates according to cancer sites in our patient sample. However, cancer site became no longer significant after the introduction of other cancer-related clinical variables. This phenomenon may suggest that physical and psychological characteristics associated with a specific type of cancer, such as poor performance status and depression, as shown in this study, rather than the cancer per se, are the main determinants of risk of suicidality of a cancer patient.

The statistically significant association between metastatic cancer and SI in this study is consistent with two existing studies [44, 45], which reported that risk of suicide was highest among cancer patients with a non-localized cancer or poor prognosis. This finding is expected because late-stage cancer is more lethal and less treatable than early-stage cancer and studies have shown that patients with terminal cancer have the highest level of hopelessness [46], a powerful predictor of suicidality in cancer patients [3, 47]. Impaired physical functioning was frequently reported as a risk factor for suicidality of cancer patients [3, 6]. In accordance with these earlier reports, we found poor performance status was significantly associated with SI in this study. Perhaps, patients with difficulties in vital functions, like eating and self-care ability, experience higher level of hopelessness/helplessness and therefore are at elevated risk for SI [33].

Previous studies seldom explored the association between treatment regimen and risk of SI during active treatment [6], mainly because most of their samples are discharged cancer survivors. Due to the Chinese cultural traditions about life and death, insufficient financial input from the government, and the lack of nursing professionals, there is very few end-of-life care institutions in contemporary China and most palliative care services are provided in large hospitals [48]. The current study compared risk of SI among patients receiving different treatment regimens and found patients receiving palliative care were at greater risk for SI relative to those receiving chemotherapy or radiotherapy. Because patients under palliative care often have a lot physical and behavioral symptoms such as intolerable pain, fatigue and insomnia [49], it is understandable that these patients have high desire for suicide or hastened death. Nevertheless, surgery was additionally found to be another robust correlate for SI in this study, with an OR of 6.62. We consider that SI is more likely to be a behavioral symptom of stress response to the surgical injury immediately after the surgery treatment [50], thereby leading to the strong association between surgery and SI.

This study has several limitations. First, the survey was conducted among tertiary general hospitals only; secondary general hospitals and tumor specialty hospitals were not included. Therefore, findings from the present study could not be generalized to cancer patients of other types of hospitals. Second, as shown in this study, the prevalence of SI varied significantly with cancer sites. Our estimation on SI prevalence might be unstable, as it can differ among different samples consisting of patients with different types of cancer. However, because our multivariable logistic regression analysis showed that the psychological and cancer-related clinical characteristics, not cancer sites, were significantly associated with SI in patients. We still can rely on these psychological and clinical characteristics to assess the risk of suicidality of an individual cancer patient. Third, social support, cancer-related stigma, and time since patients were informed the cancer diagnosis may also play some roles in the etiology of SI. Because of the limitation of study design, the present study did not collect data on these variables. More research is needed to examine the association between SI and these variables in cancer patients. Fourth, considering that patients with more severe illness (i.e., poorer performance status and late-stage cancer) were more likely to have SI and over one-fourth non-completers were “too ill to be interviewed” in this study, the selection bias from our exclusion of too ill subjects may result in underestimate the SI prevalence. Fifth, other types of non-fatal suicidal behaviors, suicide plan and attempted suicide, which are more useful for assessing suicide risk of cancer patients, have not been investigated in this study. More studies are also warranted to explore this issue. Finally, owing to the cross-sectional design, the causality between SI and these identified correlates in this study needs to be further examined in longitudinal studies.

In summary, SI is very prevalent among cancer inpatients of Chinese large general hospitals, indicating the potentially high risk of suicide of Chinese cancer inpatients. There is an urgent need for health policymakers and healthcare providers to improve early identification of high-suicide-risk patients and increase the access to psychological and psychiatric treatments in Chinese large general hospitals. Suicide prevention efforts for cancer inpatients may be useful to target on those who have depression, anxiety, severe pain, metastatic cancer, and poor performance status, and are receiving surgery and palliative care. Services for cancer inpatients should include periodic evaluation of suicidality, effective pain management, psychooncological supports, and, when necessary, psychiatric treatment and crisis intervention.

## MATERIALS AND METHODS

### Subjects

This cross-sectional survey took place in inpatient departments of two tertiary general hospitals (the Fourth Center Hospital and the People's Hospital) of a metropolitan city in northern China, Tianjin. The two hospitals are located in the two most populous districts of the city (*Heping* and *Jiangbei*) and provide various medical services to a geographically defined area of over 1.2 million people. In China, tertiary hospital is the largest-sized and highest-level hospital. It has at least 500 inpatient beds, integrates the best comprehensive medical services and is equipped with the most advanced medical equipment and technologies.

Cancer patients, who were admitted in the two hospitals between February and December 2015, were consecutively recruited. Inclusion criteria were as follows: (a) awareness of the diagnosis of cancer, which was ascertained by histological examination, regardless of sites and stages of cancer, (b) age of 18 years or older, and (c) capacity to provide informed consent. Patients were excluded if they were too ill, had cognitive impairments (i.e., dementia and delirium), or had difficulties in communication. Because several patients had not been told about the true status of their malignant tumors by their family members, and we had to collect some cancer-related variables from cancer patients per se, we only included patients who were aware of their cancer diagnoses. During the study period, a total of 735 cancer patients fulfilled the inclusion criteria and all were invited to join the survey. Among them, 69 did not complete the questionnaire, 75 refused to participate, 58 were too ill to communicate with our investigators, 10 had cognitive disorders, and 6 were ineligible for other reasons, leaving 517 completed the survey.

This study was approved by the Institutional review Board of Tianjin Anning Hospital. All participants provided written informed consent. For patients identified as having high risk of suicide, psychiatric consultation suggestions were provided to their treating oncologists.

### Measures and procedures

Candidate correlates of SI examined in this study fell into three broad domains: sociodemographic variables, general risk factors for suicidality in the general population, and cancer-related clinical characteristics.

Sociodemographic characteristics investigated included sex, age, education, marital status, place of abode (urban vs. rural), living arrangement (alone, with family members, and with others), religious belief, and perceived family economic status (poor, moderate, and good).

We assessed the presence of five general risk factors: personal history of mental illness, family history of mental illness, exposure to suicide of a family member or friend, depression and anxiety. We used the Hospital Anxiety and Depression Scale (HADS) [51] to assess the severity of depressive and anxiety symptoms of cancer patients. This 14-item scale has two subscales: seven items for depression and another seven for anxiety. Each item is rated on a 0-3 scale, yielding a total score ranging between from 0 to 21 for each subscale. Higher scores indicates more symptoms of depression or anxiety. Scores of ≥9 on the depression subscale and ≥6 on the anxiety subscale were used to denote the presence of clinically significantly depressive and anxiety symptoms in Chinese patients with physical illness, respectively [52]. The Chinese version of the HADS is reliable and valid in physically ill patients [52].

Cancer-related clinical variables included sites, stages, pain intensity, time after the diagnosis of cancer, total number of hospital admissions, functional status and cancer treatment. Given the variety of types of cancer in our study sample, cancer stage was measured by a local/regional/metastatic staging system [53]. Intensity of pain was assessed by using a simple four-point Verbal Rating Scale [54], that is, patients were required to rate their pain intensity in the past month choosing from the following descriptors: none, mild, moderate, and severe. The ECOG Performance Status Scale was used to assess how cancer affects the daily living abilities of the patient and its Chinese version has been proved to be valid and reliable [55]. The ECOG scale evaluates functional status on a scale of 0 (fully active) to 5 (dead) with higher scores denoting poorer function.

The primary outcome of this study, one-month SI, was assessed with one question asking respondents “In the past month, did you think about ending your life?”. An affirmative answer indicated the presence of SI. This question on SI was also used in many previous studies [36, 56–59].

All patients independently and anonymously completed the questionnaires on sociodemographic characteristics and SI. Trained investigators were assigned to read out questions for subjects who had difficulties in filling the questionnaires. Cancer-related clinical data were obtained by a careful review of medical records and patient interview when necessary.

### Data analysis

Prevalence of SI was calculated. Chi-square test was adopted to compare SI rates between subjects of different characteristics. Statistically significant variables in the Chi-square tests were then entered together in multivariable logistic regression with a backward stepwise entry approach to identify correlates of SI in cancer inpatients. Odds ratios (ORs) and 95% confidence intervals (CIs) were used to quantify the associations between factors and SI. The statistical significance level was set at p<0.05 (two-sided). SPSS software version 12.0 package was used for analyses.
